# Colorful Image Colorization with Classification and Asymmetric Feature Fusion

**DOI:** 10.3390/s22208010

**Published:** 2022-10-20

**Authors:** Zhiyuan Wang, Yi Yu, Daqun Li, Yuanyuan Wan, Mingyang Li

**Affiliations:** 1 Changchun Institute of Optics, Fine Mechanics and Physics, Chinese Academy of Sciences, Changchun 130033, China; 2 University of Chinese Academy of Sciences, Beijing 100049, China

**Keywords:** colorization, category conversion module, category balance module, U-Net, classification subnetwork, asymmetric feature fusion

## Abstract

An automatic colorization algorithm can convert a grayscale image to a colorful image using regression loss functions or classification loss functions. However, the regression loss function leads to brown results, while the classification loss function leads to the problem of color overflow and the computation of the color categories and balance weights of the ground truth required for the weighted classification loss is too large. In this paper, we propose a new method to compute color categories and balance the weights of color images. In this paper, we propose a new method to compute color categories and balance weights of color images. Furthermore, we propose a U-Net-based colorization network. First, we propose a category conversion module and a category balance module to obtain the color categories and to balance weights, which dramatically reduces the training time. Second, we construct a classification subnetwork to constrain the colorization network with category loss, which improves the colorization accuracy and saturation. Finally, we introduce an asymmetric feature fusion (AFF) module to fuse the multiscale features, which effectively prevents color overflow and improves the colorization effect. The experiments show that our colorization network has peak signal-to-noise ratio (PSNR) and structure similarity index measure (SSIM) metrics of 25.8803 and 0.9368, respectively, for the ImageNet dataset. As compared with existing algorithms, our algorithm produces colorful images with vivid colors, no significant color overflow, and higher saturation.

## 1. Introduction

Colorization has played an important role in processing grayscale pictures such as medical pictures, night vision pictures, electron microscopic pictures, satellite remote sensing pictures, and old photos. However, colorization is a complex and diverse problem, since the same piece of clothing can be red, blue, brown, or other colors. Therefore, it currently remains a challenging subject.

**Figure 1:**
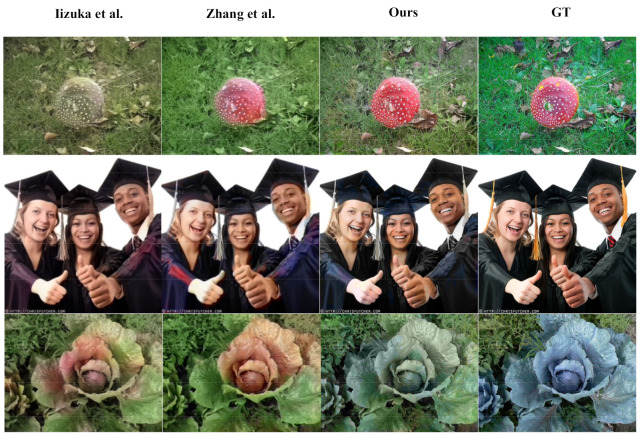
Problems with the current colorization networks. Using regression loss functions (such as Iizuka et al. [11]) results in a brownish, unsaturated result. Using classification loss functions (such as Zhang et al. [22]) results in color overflow.

Traditional colorization methods are mainly divided into two types: color expansion through adjacent pixels [1,2,3,4] and color transfer through reference images [5,6,7,8]. However, both methods require a lot of manual interaction and rely heavily on the accuracy of color marking or the selection of reference maps. In recent years, with the rapid development of deep learning, a large number of automatic colorization algorithms based on convolutional neural networks (CNNs) have been proposed. However, most colorization algorithms use regression loss functions (such as L_1_ and L_2_) [9,10,11,12,13,14,15,16,17,18,19,20,21]. These algorithms resolve the features of grayscale images and add color channels to achieve colorization. The generated colorful images have been relatively satisfactory, but the problem of brown and unsaturated generated images has persisted, as shown in Figure 1. To generate vibrant and saturated colorful images, Zhang et al. [22] used the classification loss function for colorization. However, this algorithm triggered very serious color overflow, as shown in Figure 1. Moreover, the long training time of his network made it difficult to train.

In order to improve the brown and unsaturated phenomenon of generated images, suppress the color overflow of generated images and reduce the training time of classification loss function network, we propose a new method to compute color categories and balance weights of color images. Furthermore, we propose a colorization network based on U-Net [23]. First, we propose a category conversion module and a category balance module to obtain the color categories and to balance weights. These two modules replace the original point-by-point calculation by matrix indexing, which significantly reduces the training time. Second, in order to obtain richer global features for the colorization network, we construct a classification subnetwork which classifies grayscale images according to 1000 image categories of the ImageNet dataset. The classification subnetwork constrains the colorization network with category loss to improve the colorization accuracy and saturation. Finally, inspired by Cho [24], we introduce an AFF module to fuse the multiscale features. Multiscale feature fusion enables the colorization network to grasp both global features and local features, which effectively prevents color overflow and improves the colorization effect. As a result, our colorization algorithm produces vibrant images with no visible color overflow. The contributions of this work are:

1. A category conversion module and a category balance module are proposed to significantly reduce the training time.
2. A classification subnetwork is proposed to improve colorization accuracy and saturation.
3. An AFF module is introduced to prevent color overflow and to improve the colorization effect.

## 2. Related Work

### 2.1. Traditional Colorization Method

Traditional colorization methods require manual interaction. They are divided into two types: color expansion through adjacent pixel points and color transfer through reference pictures.

#### 2.1.1. Color Expansion

The color expansion method was proposed by Levin et al. [1]. This work pointed out that two neighboring pixel points with similar grayscale values have similar color and based on this, the manually labeled colored lines were expanded to the whole image. On the basis of the abovementioned finding, Yatziv et al. [2] added a weighted distance function between pixels to guide colorization. Qu et al. [3] and Luan et al. [4] used image texture feature similarity to reduce the computational complexity. The color expansion method generates color-symbolic images as expected, but color confusion occurs due to inaccurate, manually labeled colored lines or at the edges of the image.

#### 2.1.2. Color Transfer

The color transfer method was proposed by Welsh et al. [5]. This work selected color pictures similar to grayscale pictures as reference pictures, and transferred the colors of reference pictures to pixel points with similar grayscale values in grayscale pictures. Based on this, Irony et al. [6] cut high-resolution reference pictures and transferred the color of the reference pictures based on texture features. To solve the problem that reference pictures are not easily accessible, Liu et al. [7] searched the internet for color pictures similar to grayscale pictures. Wang et al. [8] searched for the color pictures with the highest similarity as reference pictures through the semantics of grayscale pictures. The color transfer method reduces some manual operations, but the colorization effect depends on the reference picture and the selection of the color transfer method.

### 2.2. Deep Learning-Based Colorization Algorithms

Deep learning-based colorization algorithms enable end-to-end automatic colorization. According to the loss function of colorization, they are divided into two types: regression loss function and classification loss function.

#### 2.2.1. Regression Loss Function

The vast majority of colorization algorithms [9,10,11,12,13,14,15,16,17,18,19,20,21] use regression loss functions. Cheng et al. [9] extracted image features using a CNN and combined bilateral filtering to enhance colorization. Larsson et al. [10] used a very deep convolutional network (VGG) to obtain the semantics of an image and guided colorization based on the hue and chroma histogram of each pixel point. Iizuka et al. [11] constructed a two-channel CNN to extract global and local features of the image separately, to fuse the two features, and to add scene classification labels to improve the colorization effect. Nazeri et al. [12] constructed conditional generative adversarial networks (cGANs) to build colorization networks. Patricia et al. [15] constructed a two-channel ChromaGAN to output the category distribution and the generated color images, and introduced category distribution of the images to enhance the colorization effect. Su et al. [19] cropped the objects in the image, constructed a multichannel CNN to color each object of the crop and the overall image, and fused multiple color images according to the weights to improve the colorization effect. Wu et al. [20] used GANs to generate color images associated with grayscale images to guide the colorization of grayscale images. Jin et al. [21] constructed a three-channel HistoryNet that contained image category, semantics, and colorization, using categorical and semantic information to guide colorization. These algorithms achieved the desired colorization results. However, due to the uncertainty and diversity of image colorization, regression loss functions assigned each object’s color to the sum of all its possible colors. This eventually resulted in brown and unsaturated colorization effect.

#### 2.2.2. Classification Loss Function

Only Zhang et al. [22] used classification loss function of colorization. In order to use classification loss function, this work constructed 313 color categories according to the pixel *a* and *b* values. To calculate the color category of each pixel point in a color image, Zhang et al. calculated the geometric distance between each pixel point *a* and *b* value and its 32 closest color categories *a* and *b* values. Next, the color category probability distribution of each pixel was obtained by Gaussian weighting, and the color category with the highest probability was selected. Finally, to make the colorization vivid, this work balanced the weights using the color category probability distribution of the ImageNet training set. The color categories and balance weights were formulated as follows:

(1) $$Z_{h,w,q}=e^{-d^{2}/2\sigma^{2}}/\sum_{i=0}^{31}e^{-d_{i}^{2}/2\sigma^{2}}$$

(2) $$\omega (Z_{h,w})=w_{q^{*}},where\;q^{*}=\arg m\underset{q}{a}xZ_{h,w,q}$$

(3) $$w\propto {\left((1-\lambda )\tilde{p}+\frac{\lambda }{Q}\right)}^{-1}$$

(4) $$E\left[w\right]=\sum_{q}{\tilde{p}}_{q}w_{q}=1$$

where *d* is the geometric distance between pixel point *a* and *b* values and its 32 closest color categories *a* and *b* values; *h* and *w* are the positions of the pixel; *q* is the color category of the pixel; σ is the Gaussian kernel with Gaussian weighting, which is taken as 0.5 here; $\tilde{p}$ is the color category distribution of all pixels in the ImageNet training set images; Q represents the number of color categories used, which is 313 in this study; λ represents the weight of mixing the average distribution of each color category and the color category distribution of the ImageNet training set of 1.28 million images, and 0.5 was tested to be the most effective. However, this method lead to long training time and training difficulties for the colorization network due to the large amount of computation. Moreover, although this work generated vibrant and vivid color images, it resulted in severe color overflow because the colorization network of this work did not fuse global features and local features of the input image.

## 3. Method

### 3.1. Overview

**Figure 2:**
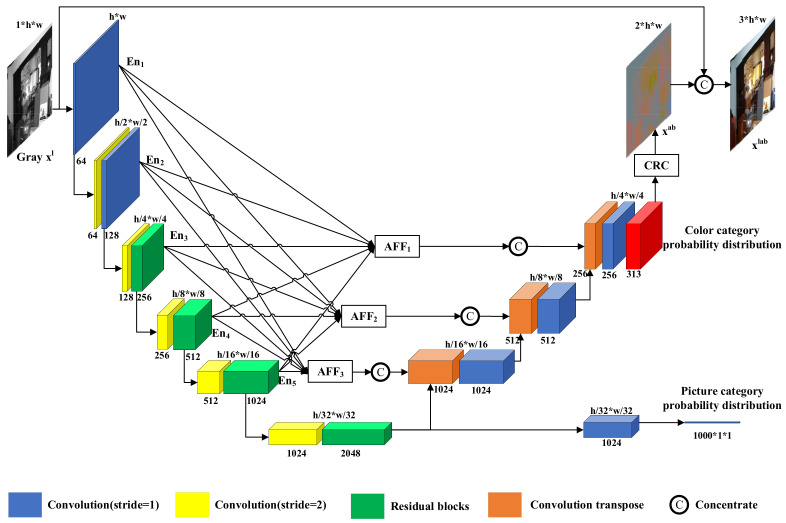
Network structure. Our colorization network consists of an encoder (left), a classification subnetwork (bottom right), a decoder (right), three AFF modules and a CRC module.

Given a grayscale image $x^{l}\in R^{1*h*w}$ as input, the purpose of colorization is to predict the remaining a and b channels $x^{ab}\in R^{2*h*w}$ in the Lab channel and turn the single channel $x^{l}$ into a three-channel color image $x^{lab}\in R^{3*h*w}$; l, a and b represent the brightness of the Lab color space, and range from red to green and from yellow to blue, respectively. In this work, we design an end-to-end colorization network based on U-Net. As shown in Figure 2, our colorization network consists of three parts: an encoder, a classification subnetwork, and a decoder. Our colorization network outputs the picture category probability distribution and color category probability distribution. The color category probability distribution becomes $x^{ab}$ after the color recovery (CRC) module $x^{ab}$ concentrates $x^{l}$ to obtain the colorful image $x^{lab}$. 

As shown in Figure 2, the encoder consists of six layers of convolutional blocks. When input $M_{in}\in R^{n*c*h*w}$ passes through the convolution block, the obtained detailed features $M_{out}\in R^{n*2c*h/2*w/2}$ are saved and passed to the next layer of the convolution block. After six layers of convolutional blocks feature extracting, the encoder generates global features $x^{g}\in R^{2048*h/32*w/32}$ of input grayscale images $x^{l}\in R^{1*h*w}$. The classification subnetwork consists of a convolution module and an average pooling layer. The classification subnetwork resolves the global features $x^{g}\in R^{2048*h/32*w/32}$ generated by the encoder into the picture category probability distribution $\hat{Y}\in R^{n*1000*1*1}$. The decoder consists of three layers of convolutional blocks. Before input $M_{in}\in R^{n*c*h*w}$ passes through the convolutional block, it is concatenated with the same size features of the AFF module output. The decoder resolves the global features $x^{g}\in R^{2048*h/32*w/32}$ generated by the encoder into color class probability distributions $\hat{Z}\in R^{n*313*h/4*w/4}$ of the grayscale image $x^{l}$.

### 3.2. Calculating Color Categories and Balance Weights

In order to reduce the computation of color categories and balance weights, we propose a category conversion module and a category balance module. These two modules obtain the color categories and balance the weights of real colorful images for training.

#### 3.2.1. Category Conversion Module

**Figure 3:**
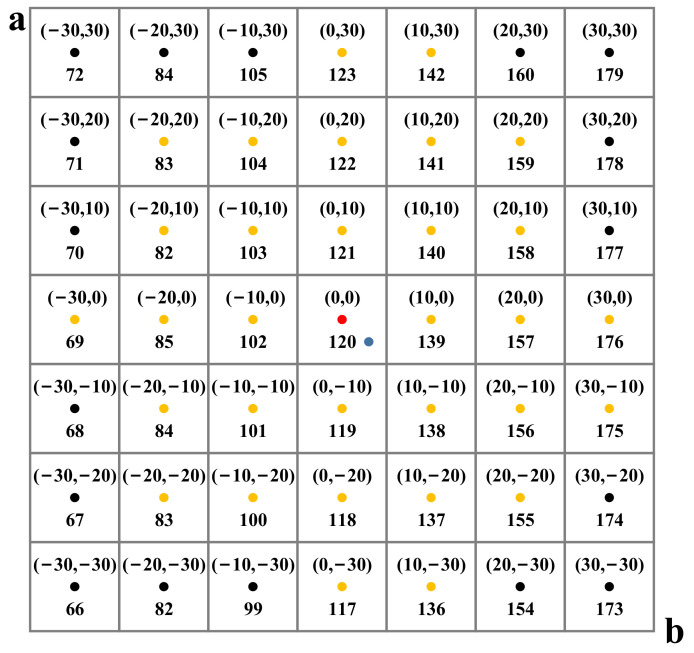
Part of the color category distribution. The lower half values of the small squares are their corresponding color categories q. For the pixel (blue dot) $\left(3,-3\right)$, the same color category 120 is obtained for the method of Zhang et al. and our method.

As shown in Figure 3, given the pixel (blue dot) with a and b values (3, −3), Zhang et al. [22] calculated the Euclidean distances d between the blue dot and the 32 nearest color categories (red and yellow dots) to the blue dot. Next, they obtained the probability distribution of each color category by Gaussian weighting using Equation (1). Finally, they selected the color category with the highest probability 120 using Equation (2). Equation (1) decreases monotonically with d, so the color category of the pixel $\left(a,b\right)$ is the color category q, corresponding to the center point $\left(a_{0},b_{0}\right)$ of the small square where the pixel point is located.

Therefore, in order to obtain the color category of pixel $\left(a,b\right)$, we calculated the $\left(a_{0},b_{0}\right)$ value of the center point of the 10 × 10 square where the pixel $\left(a,b\right)$ was located. Next, we converted $\left(a_{0},b_{0}\right)$ to the corresponding color category q. As shown in Figure 3, given the pixel $\left(3,-3\right)$, we calculated the values $\left(0,0\right)$ for the center point (red dot) of the small square where this pixel was located and determine the color category 120 for $\left(3,-3\right)$ by the color category 120 corresponding to $\left(0,0\right)$.

To calculate the color categories $Z\in R^{n*h*w}$ corresponding to the ground truth a and b channels $x^{ab}\in R^{n*2*h*w}$, we used the above method to construct the color category matrix M indexing the color category Z through $Z=M\left(x^{ab}\right)$, where n is the batch size for one training and h and w are the pixel locations. The color category matrix $M\in R^{420}$ is formulated as follows:

(5) $$M\left[22*\left(a_{0}/10+9\right)+\left(b_{0}/10+11\right)\right]=q\left(a_{0},b_{0}\right)$$

(6) M[k]=−1,where k is the element without a index value

where [] is an integer symbol, $q\left(a_{0},b_{0}\right)$ is the color class q corresponding to $\left(a_{0},b_{0}\right)$. 

The category conversion module calculates $a_{0}$ and $b_{0}$ values of real, colorful pictures *a* and *b* channels $x^{ab}\in R^{n*2*h*w}$ and indexes the corresponding color categories $Z\in R^{n*h*w}$ by color category matrix. The color categories $Z\in R^{n*h*w}$ are formulated as follows:

(7) $$x^{ab}=\left[x^{ab}/10+0.5\right]$$

(8) $$Z=M\left(\left(x^{ab}\left[:,0,:,:\right]+9\right)*22+\left(x^{ab}\left[:,1,:,:\right]+11\right)\right)$$

#### 3.2.2. Category Balance Module

In real colorful pictures, since the backgrounds such as sky, grass, ocean, and walls occupy a large number of pixels, most of the pixels are color categories with low values of *a* and *b*. To encourage diversity in colorization, we construct the balance weight matrix ω, which is formulated as follows:

(9) $$w={\left(\left(1-\lambda \right)\tilde{p}+\lambda /Q\right)}^{-1}$$

(10) $$\omega =\frac{w^{-1}}{\sum_{q}{\tilde{p}}_{q}w_{q}{}^{-1}}$$

where Q represents the number of color categories used, here is 313; λ represents the weight of mixing the average distribution of each color category and the color category distribution of the ImageNet training set of 1.28 million images, and 0.5 was set. The category balance module obtains the corresponding balance weight $\omega (Z_{h,w})$ based on the color category $Z_{h,w}$. Finally, the category conversion module and the category balance module are formulated as follows:

(11) $$Z,\omega \left(Z_{h,w}\right)=H\left(x^{ab}\right)$$

### 3.3. Residual Block

**Figure 4:**
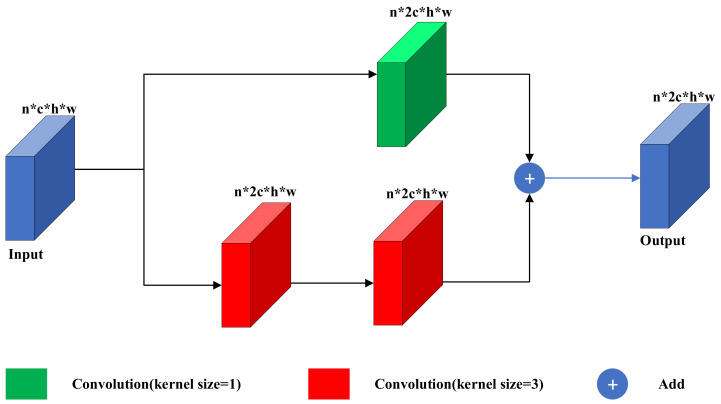
The structure of the residual block in the green part of Figure 2. Our residual block consists of one 1 × 1 convolution kernel and two 3 × 3 convolution kernels.

In order to solve the problem of training difficulties brought by the deeper layers of the colorization network, we construct the residual block based on the idea of ResNet [25]. As shown in Figure 4, our residual block consists of one 1 × 1 convolution kernel on the top and two 3 × 3 convolution kernels on the bottom. The upper convolution kernel only transforms the number of input channel to the output, and the lower convolution kernels transform the number of input channel and extract the features. The summation of upper and lower features optimizes the forward path of the colorization network and makes the network easier to train. Therefore, our residual block can effectively solve the problem of network degradation brought by the deeper layers of the network.

### 3.4. Asymmetric Feature Fusion Module

In most U-Net-based algorithms, the decoder only concatenates features of the same scale as the encoder. However, the top-down downsampling structure of the encoder causes only the high scale features to act on the low scale features, so the high scale features concatenated by the decoder are not affected by the low scale features, resulting in the degradation of the colorization effect.

**Figure 5:**
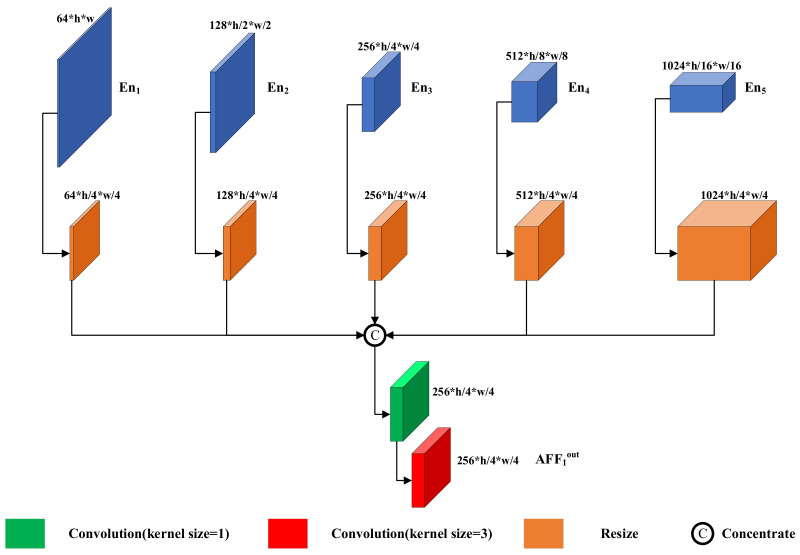
Asymmetric feature fusion module structure. The AFF module consists of resized modules, a 1 × 1 convolution kernel, and a 3 × 3 convolution kernel.

Inspired by multi-input multioutput U-Net (MIMO-UNet) [24] and dense connections between intra-scale features [26], we introduce the AFF module, as shown in Figure 5. 

The AFF module concatenates the features of all scales of the encoder $\left(En_{1}-En_{5}\right)$, outputs the multiscale fused features with the convolution kernel, and finally concatenates the features of the corresponding scales with the decoder. Three AFFs $\left(AFF_{1},AFF_{2},AFF_{3}\right)$ are formulated as follows:

(12) $$AFF_{1}^{out}=AFF_{1}\left(Subs_{4}\left(En_{1}\right),Subs_{2}\left(En_{2}\right),En_{3},Ups_{2}\left(En_{4}\right),Ups_{4}\left(En_{5}\right)\right)$$

(13) $$AFF_{2}^{out}=AFF_{2}\left(Subs_{8}\left(En_{1}\right),Subs_{4}\left(En_{2}\right),Subs_{2}\left(En_{3}\right),En_{4},Ups_{2}\left(En_{5}\right)\right)$$

(14) $$AFF_{3}^{out}=AFF_{3}\left(Subs_{16}\left(En_{1}\right),Subs_{8}\left(En_{2}\right),Subs_{4}\left(En_{3}\right),Subs_{2}\left(En_{4}\right),En_{5}\right)$$

where $AFF_{n}^{out}$ denotes the output of the nth layer, $En_{n}$ denotes the output of the nth convolutional block of the encoder, $Subs_{k}$ denotes downsampling by a factor of k, and $Ups_{k}$ denotes upsampling by a factor of k.

### 3.5. Color Recovery Module

We construct the inverse color category matrix $M^{-1}$ indexing the values of a and b through $x_{0}=M^{-1}\left(q\right)$, where q is the color category of pixel and $M^{-1}$ is the inverse of the color category matrix M. The index of $M^{-1}$ is the color category q, corresponding to $\left(a_{0},b_{0}\right)$ of q. 

The color recovery module divides the color class distribution $\hat{Z}\in R^{313*h/4*w/4}$ by the annealing parameter and selects the color category with the highest probability. Next, we use $M^{-1}$ to index the $\left(a,b\right)$ value $x_{0}\in R^{2*h/4*w/4}$. Finally, we upsample $x_{0}$ by a factor of 4 to obtain $x_{ab}\in R^{2*h*w}$. The color recovery module is formulated as follows:

(15) $$q^{*}=\arg\underset{q}{\max}\left({\hat{Z}}_{h,w,q}/T\right)$$

(16) $$x_{0}=M^{-1}\left(q^{*}\right)$$

(17) $$x^{ab}=Ups_{4}\left(x_{0}\right)$$

*T* is the annealing parameter, which is taken as 0.38 here. $Ups_{k}$ denotes the upsampling amplification *k* times.

### 3.6. Colorization with Classification

Although the classification loss function can generate vibrant colors, the colorization inaccuracy caused by not obtaining the global environment of the input grayscale image is always present. To solve this problem, we construct a classification subnetwork and facilitate the optimization by also training for picture category losses jointly with color category losses. The classification subnetwork resolves the global features $x^{g}\in R^{2048*h/32*w/32}$ acquired by the encoder into the picture category probability distribution $\hat{Y}\in R^{n*1000*1*1}$ for grayscale images. We use 1000 category labels m∈[0,999] delineated by the ImageNet dataset, which cover all objects in the natural and human world. The classification subnetwork makes the global features of the encoder output more comprehensive through the picture category loss function, thus, enabling the decoder to resolve more accurate color categories. The classification network uses the cross-entropy loss function and is formulated as follows:

(18) $$L_{cls}\left(Y,\hat{Y}\right)=\sum_{h,w}\sum_{m}Y_{h,w,m}\log\left({\hat{Y}}_{h,w,m}\right)$$

where $Y_{h,w,m}\in R^{n*1*1}$ is the category label of the real image. The decoder outputs the color category probability distribution $\hat{Z}\in R^{n*313*h/4*w/4}$ of the grayscale image. The colorization network uses the cross-entropy loss function and is formulated as follows:

(19) $$L_{col}\left(Z,\hat{Z}\right)=\sum_{h,w}\omega \left(Z_{h,w}\right)\sum_{q}Z_{h,w,q}\log\left({\hat{Z}}_{h,w,q}\right)$$

where $Z,\omega \left(Z_{h,w}\right)$ is the color category and balance weight of the real image, which can be obtained by the category conversion module and the category balance module. The total loss function is formulated as follows:

(20) $$L=\lambda_{col}L_{col}+\lambda_{cls}L_{cls}$$

where $\lambda_{col}$ and $\lambda_{cls}$ are hyperparameters controlling the picture category loss and color category loss.

## 4. Experiments

### 4.1. Experimental Details

To verify the effectiveness of our proposed colorization algorithm, we built the colorization network in the pytorch framework and trained it with two NVIDIA GeForce RTX 3090 graphics cards. In this experiment, approximately 1.28 million images containing 1000 image categories from the ImageNet training set were used to train the colorization network, and 50,000 images of the ImageNet validation set were used to test the colorization effect.

We initialized our colorization network with the Xavier normal function and trained the colorization network with the SGD optimizer. The initial learning rate, momentum parameter, and weight decay were set to 10^−3^, 0.9, and 10^−4^, respectively. The learning rate decays gradually with training, and $\lambda_{col}$ and $\lambda_{cls}$ are set to 1 and 0.003, respectively. Batch size is set to 64 and the input image size is fixed to 224 × 224. Our colorization network is trained for 10 epochs and the training time for each epoch is approximately 16 h. The learning rate change is formulated as follows:

(21) $$lrIter=lr*\alpha_{1}+lr*\alpha_{2}/100$$

(22) $$\alpha_{1}=EpochIter/\left(EpochNum*EpochLength\right)$$

(23) $$\alpha_{2}={\left(1-\alpha_{1}\right)}^{lrPow}$$

where EpochNum is the number of training epochs; EpochLength is the total number of training epochs; EpochIter is the current number of training; lrPow is the exponential parameter, here is 0.9; lrIter is the current learning rate; and lr is the initial learning rate.

### 4.2. Calculating Time Experiments

To verify the accuracy of calculating the color categories and balance weights of color images proposed in this paper, we randomly selected 200 images from each image category of the ImageNet training set of 1000 image categories (1,281,167 images in total) and calculated the color categories and corresponding balance weights of the images using Zhang et al.’s method [22] and our method for 200,000 images, respectively. For approximately 43.9 billion pixels of 200,000 images, the color categories and corresponding balance weights calculated by the two methods are exactly the same. However, as shown in Table 1, the method of Zhang et al. takes approximately 3 days of computation in our computer, while our method takes less than 2 h of computation.

The batch size of our colorization network is 64, and therefore, training a batch requires computing the color categories and corresponding balance weights for 64 images with a resolution of 224 × 224. As shown in Table 1, computing the color categories and balance weights for approximately 3.2 million pixels on our computer takes about 18.86 s for Zhang et al.’s method, while our method takes only approximately 0.4 s.

### 4.3. Quantitative Analysis

In order to quantitatively evaluate the colorization effect of our colorization network, we use the SSIM and the PSNR as the evaluation indexes for quantitative analysis.

The SSIM evaluates the similarity between a color picture generated by the colorization network and a real picture in terms of brightness, contrast, and structure. The SSIM can sensitively perceive the local structural differences between the two pictures. The SSIM takes values from 0 to 1, and a larger SSIM value means that the two images are more similar. SSIM is formulated as follows:

(24) $$l\left(x,y\right)=\left(2\mu_{x}\mu_{y}+C_{1}\right)/\left(\mu_{x}{}^{2}+\mu_{y}{}^{2}+C_{1}\right)$$

(25) $$c\left(x,y\right)=\left(2\sigma_{x}\sigma_{y}+C_{2}\right)/\left(\sigma_{x}{}^{2}+\sigma_{y}{}^{2}+C_{2}\right)$$

(26) $$s\left(x,y\right)=\left(\sigma_{xy}+C_{3}\right)/\left(\sigma_{x}\sigma_{y}+C_{3}\right)$$

(27) $$SSIM\left(x,y\right)=l{\left(x,y\right)}^{\alpha }*c{\left(x,y\right)}^{\beta }*s{\left(x,y\right)}^{\gamma }$$

where $\mu_{x}$ and $\mu_{y}$ denote the mean of image *x* and *y*, respectively; $\sigma_{x}$ and $\sigma_{y}$ denote the variance of image *x* and *y*, respectively; $\sigma_{xy}$ denotes the covariance of image *x* and *y*; $C_{1},C_{2},C_{3}$ are constants; and α,β,γ denote the importance of each module.

The PSNR is an objective measure of image quality evaluation before and after image compression. The larger the value of PSNR, the less distorted the image. The PSNR of a real image *x* with resolution *m* × *n* and a generated image *y* is calculated as follows:

(28) $$MSE=\frac{1}{mn}\sum_{i=0}^{m-1}\sum_{j=0}^{n-1}{\left[x\left(i,j\right)-y\left(i,j\right)\right]}^{2}$$

(29) $$PSNR=10*\log_{10}\left(MAX_{x}^{2}/MSE\right)$$

where $MAX_{x}^{2}$ indicates the maximum possible pixel value of the image.

We tested our algorithm on 50,000 images from the ImageNet validation set against the algorithms of Larsson et al. [10], Iizuka et al. [11], Zhang et al. [22], Deoldify [18], and Su et al. [19]. Table 2 shows the comparison of our experimental results with the SSIM and the PSNR of the above algorithms. It can be clearly seen that our colorization network has higher SSIM and PSNR values, which means the colorization effect of our network is better.

### 4.4. Qualitative Analysis

**Figure 6:**
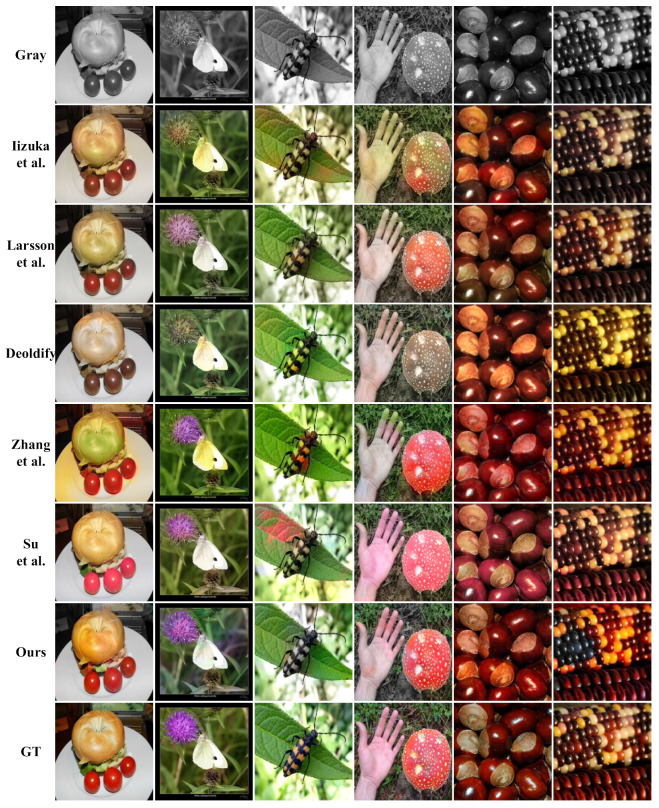
Visualization comparison of our colorization algorithm and other colorization algorithms. Our colorization network generates more vivid and saturated colorful images.

In order to verify the effectiveness of our colorization algorithm, in this paper, we compare our colorization algorithm with those of Larsson et al. [10], Iizuka et al. [11], Zhang et al. [22], Deoldify [18], and Su et al. [19]. We use 50,000 images from the ImageNet validation set for testing and adjust the resolution of the generated images to 256 × 256. The experimental results are shown in Figure 6, where our algorithm generates more vivid and more saturated colorful images.

As shown in Figure 6, our algorithm generates more vivid and saturated color images as compared with Larsson et al., Iizuka et al., Deoldify, and Su et al. Regarding the color of the small tomatoes in the first column of images, as compered with our bright red color, the other algorithms generate less saturated colors, showing a dark red or unnatural pink. In contrast to our vivid saturated purple flower, the other algorithms generate dull colors, rendering gray and mauve. In addition, as compared with Zhang et al., our algorithm effectively prevents color overflow and oversaturation. Regarding the hand in the fourth column, the fingertips of Zhang et al.’s algorithm overflow a very obvious green color and the mushroom is oversaturated with red, while our algorithm generates a more natural and vivid color for the hand and mushroom. Furthermore, our generated images successfully maintain the integrity and coherence of the color of the same object. Regarding the color of the third column of leaves, our algorithm effectively guarantees a bright green, while the algorithms of Zhang et al. and Su et al. appear unnatural red.

### 4.5. Ablation Experiments

We designed ablation experiments to demonstrate that adding a classification subnetwork and AFF module to the colorization network can effectively improve the colorization effect. We used the U-Net with the classification subnetwork and AFF module removed as the baseline network and trained it on the ImageNet 50,000 validation set. From Table 3, we can see that the PSNR and SSIM values are higher after adding the classification subnetwork and AFF module, which indicates that the classification subnetwork and AFF module can significantly improve the colorization effect of the colorization network.

**Figure 7:**
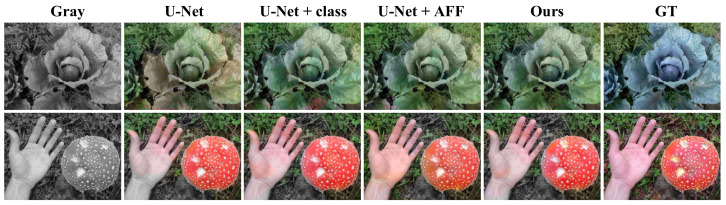
Ablation experiments. The classification subnetwork can help the colorization network to color more accurately. The AFF module can improve the color overflow phenomenon and can enhance the colorization effect.

In total, we performed three sets of ablation experiments: U-Net plus the classification subnetwork, U-Net plus the AFF module, and our colorization network. As can be seen in Table 2 as well as Figure 7, the classification subnetwork and the AFF module play a positive role in colorization.

As shown in Figure 7, the colorful images generated by U-Net have the problems of color overflow and low saturation. As for the cabbage in the first row, the color of the U-Net-generated picture leaves is gray-green, which is not bright enough and the color distribution is not uniform. After adding the classification subnetwork, the color of the leaves is a more vivid tender green, which indicates that the classification subnetwork can help the colorization network to color more accurately, but an obvious color overflow appears in the lower middle. After adding the AFF module, there is no obvious color overflow and the color of the leaves is a bright tender green, indicating that the AFF module can improve the color overflow phenomenon and enhance the colorization effect. The U-Net plus AFF module improves the color overflow phenomenon, but the color of the vegetable leaves is light. In the second row of images, the U-Net generated hand and mushroom are light in color and the tip of thumb shows color overflow. After adding the classification subnetwork, the color of hand and mushroom are more vivid, but the tip of thumb still have green color overflow. After adding the AFF module, there is no obvious color overflow, and the hands and mushrooms are healthy flesh color and bright red, respectively. It can be seen that the sorting subnetwork and AFF module can significantly improve the colorization effect.

### 4.6. User Study

**Figure 8:**
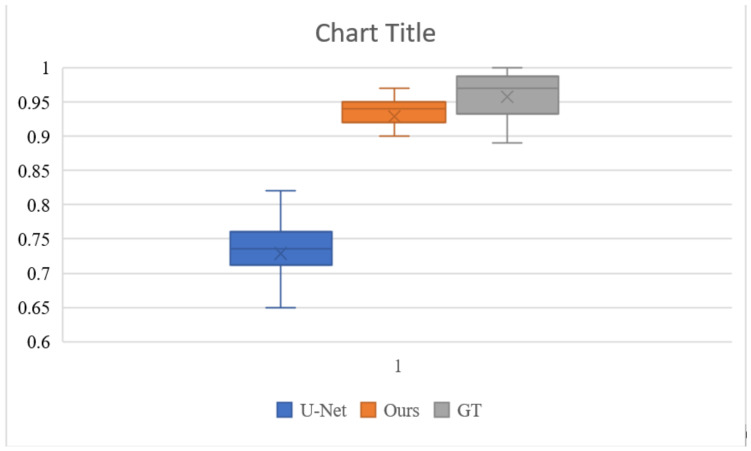
Boxplots of the naturalness of the images evaluated by different users. The 92.9% of our colorization network is closer to the 95.8% of ground truth than the 72.9% of the base U-Net. This indicates that our algorithm generates more natural color pictures.

To better evaluate the colorization effect of our algorithm, we conducted a user study to evaluate the results of the U-Net base network, the results of our colorization network, and the ground truth validation images. The study was completed by 20 participants with normal or corrected-to-normal and without color blindness. We randomly selected 100 images of different categories in the test set, for a total of 300 images. All images were displayed at a resolution of 256 × 256 pixels. Each participant was shown 300 pictures and asked to respond “Does this picture look natural?” to each picture within 1 s. Figure 8 and Table 4 show the results of the experiment. The U-Net performed poorly, with only 72.9% of the images considered to be natural. Our colorization network had 92.9% of the images considered to be natural, which was very close to the ground truth’s 95.8%. This is a good indication that our algorithm can generate more natural and saturated colors.

### 4.7. Limitation

Although our algorithm achieves better colorization results, our colorization algorithm does not determine the color category of each pixel of the input image. As shown in Figure 2, our network outputs a color category resolution of 56 × 56 instead of the input image 224 × 224, after which we obtain a color image of the corresponding resolution by upsampling 4 times. In order to obtain more accurate color categories and colorization effects, we adjust the resolution of the output color categories to the resolution of the input image 224 × 224 and train using the same dataset and training method. 

**Figure 9:**
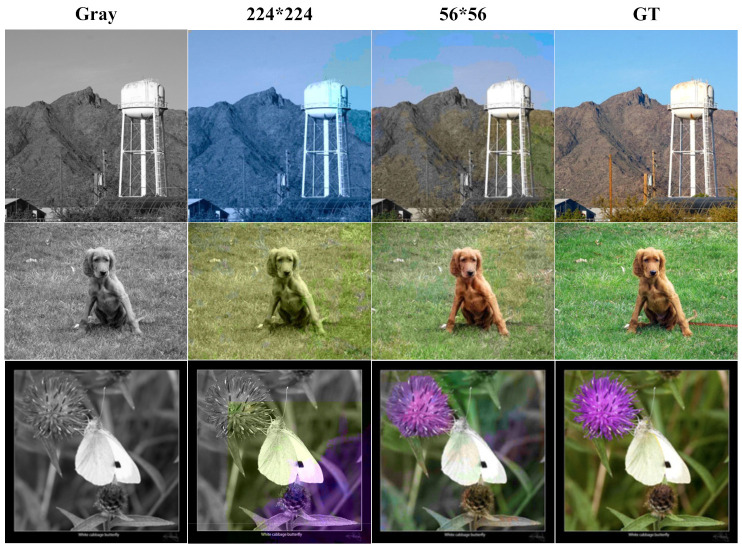
Colorization effect of pixel-level network. A single color (blue, green) fills the whole picture, and the last picture appears as an uneven block of color.

The generated color images are shown in Figure 9. The pixel-level network generates color images where a certain single color (blue, green) fills the whole image and uneven blocks of color appear. This is probably caused by two reasons. First, our classification of color categories is not accurate enough. Second, when the network becomes a pixel-level network, our network does not effectively capture the local features of the input image. In the future, we may solve this problem by dividing finer color categories or using generative adversarial networks.

## 5. Conclusions

In this paper, we propose a new method to compute color categories and balance weights of color images. Furthermore, we propose a U-Net-based colorization network incorporating a classification subnetwork and an AFF module. The category conversion module and the category balance module significantly reduce the training time. The classification subnetwork can significantly improve the colorization accuracy and saturation. The AFF module can significantly prevent color overflow and improve the colorization effect. Quantitative experiments show that our colorization network has higher PSNR and SSIM values of 25.8803 and 0.9368. Qualitative experiments show that the colorization effect of our colorization network is higher than that of existing algorithms. In addition, our improved method of calculating color categories and balance weights for color images should also attract more scholars to use color categories for colorization.

## Figures and Tables

**Table 1:** Calculation time for color categories and balance weights. The values show that our method is faster to compute.

Methods	43.9 Billion Pixels	3.2 Million Pixels
Zhang et al. [22]	3 days	18.86 s
Ours	2 h	0.4 s

**Table 2:** Quantitative analysis of colorization effect. As compared with the PSNR and SSIM values of other colorization algorithms, the colorization effect of our network is better.

Method	PSNR↑	SSIM↑
Iizuka et al. [11]	23.6362	0.9173
Larsson et al. [10]	25.1067	0.9266
Deoldify [18]	23.5372	0.9144
Zhang et al. [22]	21.7910	0.8915
Su et al. [19]	25.7440	0.9202
Ours	25.8803	0.9368

**Table 3:** Ablation experiments. The PSNR and SSIM values show that the classification subnetwork and the AFF module play a positive role in the colorization effect of the network.

Method	PSNR↑	SSIM↑
U-Net	23.1783	0.8921
U-Net + Classifier	24.1615	0.9119
U-Net + AFF	24.7595	0.9260
Ours	25.8803	0.9368

**Table 4:** Naturalness of user study. The values show that our network generates more vivid color pictures as compared with the base U-Net.

Approach	Naturalness (Median)
U-Net	72.9%
Ours	92.9%
GT	95.8%

## Data Availability

The data presented in this study are available on request from the corresponding author.
